# MiR-425-5p promotes invasion and metastasis of hepatocellular carcinoma cells through SCAI-mediated dysregulation of multiple signaling pathways

**DOI:** 10.18632/oncotarget.15958

**Published:** 2017-03-07

**Authors:** Feng Fang, Tianqiang Song, Ti Zhang, Yunlong Cui, Gewen Zhang, Qingqing Xiong

**Affiliations:** ^1^ Department of Hepatobiliary Cancer, Tianjin Medical University Cancer Institute and Hospital, Ti-Yuan-Bei, Tianjin 300060, China; ^2^ Department of Surgery, Xiangya Hospital, Central South University, Changsha 410008, Hunan, China

**Keywords:** miR-425-5p, hepatocellular carcinoma, SCAI, PTEN, integrin β1

## Abstract

MicroRNAs (miRNAs) play critical roles in hepatocellular carcinoma (HCC) progression and are key determinants of prognosis. In this study, we found that miR-425-5p was elevated in HCC and correlated with poor prognostic clinicopathological features and low post-operative long-term survival. Multivariate survival analysis indicated that miR-425-5p expression was an independent risk factor for overall and disease-free survival. Interestingly, miR-425-5p promoted invasion and metastasis by HCC cells, but not HCC cell proliferation or apoptosis *in vitro*. SCAI and PTEN were determined to be downstream targets of miR-425-5p. miR-425-5p-mediated effects were inhibited by ectopic expression of SCAI, and PTEN exhibited a smaller inhibitory effect. SCAI also suppressed PTEN expression. In addition, miR-425-5p promoted epithelial-to-mesenchymal transition (EMT), which was antagonized by SCAI. miR-425-5p also promoted HCC cell invasion and metastasis via SCAI-mediated dysregulation of integrin β1-Fak/Src-RhoA/CDC42, PTEN-AKT, and TIMP2-MMP2/MMP9 signaling. Finally, miR-425-5p promoted metastasis in a xenograft mouse model of HCC. These results indicate that miR-425-5p facilitates EMT and extracellular matrix degradation and promotes HCC metastasis through SCAI-mediated dysregulation of multiple signaling pathways. MiR-425-5p is therefore a potential prognostic biomarker and novel therapeutic target in HCC.

## INTRODUCTION

Hepatocellular carcinoma (HCC) is the second leading cause of cancer-related death in China [1]. Despite improvements in HCC therapeutic strategies, the long-term survival of patients with HCC following hepatectomy remains unsatisfactory as a result of recurrence and metastasis [2, 3]. Additionally, the molecular mechanisms underlying HCC development have not been fully elucidated [4]. A better understanding of the events responsible for HCC metastasis is critical important for prevention and treatment.

MicroRNAs (miRNAs) are highly conserved, small non-coding regulatory RNAs that are 19–22 nucleotides in length. Many human miRNAs have been identified, and aberrant expression of these miRNAs plays essential roles in various physiological and pathological processes [5]. Previous studies have demonstrated that miRNAs play critical roles in HCC progression (e.g. cell proliferation and migration, apoptosis, and metastasis) [6–10]. However, the pathological relevance and significance of the majority of miRNAs in HCC metastasis are not yet clear. In this study, we investigated the potential clinical value of miR-425-5p in HCC. In addition, we evaluated miR-425-5p function in HCC growth and metastasis using *in vitro* and *in vivo* models.

## RESULTS

### MiR-425-5p expression is elevated in human HCC tissue

**Figure 1:**
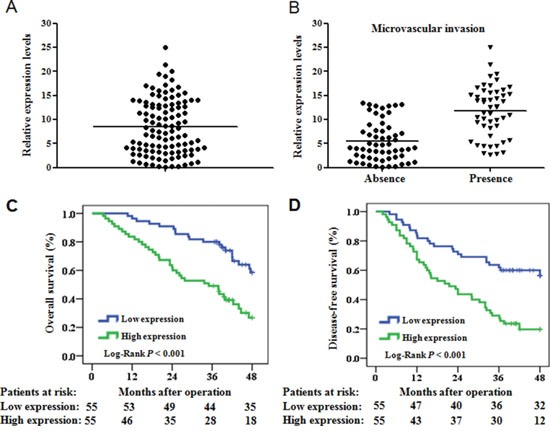
MiR-425-5p is up-regulated in HCC and is correlated with OS and DFS **(A)** Quantification of mature miR-425-5p expression in 110 paired HCC and ANLT samples using qRT-PCR. **(B)** MiR-425 expression in HCC tissue with (n = 52) or without (n = 58) microvascular invasion. Expression was compared using ANOVA. **(C** and **D)** The OS and DFS of patients with high or low miR-425-5p expression.

We evaluated miR-425-5p expression in HCC tissue and adjacent non-tumor liver tissue (ANLT) from 110 HCC patients using quantitative real-time PCR (qRT-PCR). MiR-425-5p was up-regulated relative to ANLT in 101 of 110 (91.8%) HCC cases. The median fold increase in expression was 8.9 (range: 1.1–25) (Figure 1A). Additionally, miR-425-5p expression was higher in HCC cases with than without microvascular invasion (median fold increase in expression: 12.5 vs. 5.8; *P* < 0.05) (Figure 1B).

### High miR-425-5p expression is associated with poor clinicopathological features and post-operative survival in HCC

We divided the 110 HCC patients into two groups using the median level of miR-425-5p as the cut-off value. The relationship between miR-425-5p expression and patient clinicopathological features was analyzed. High miR-425-5p expression was associated with poor clinicopathological features including tumor nodular number (*P* = 0.041), microvascular invasion (*P* = 0.022), TNM stage (*P* = 0.028), and BCLC stage (*P* = 0.004) (Table 1).

**Table 1:** Correlations between miR-425-5p expression levels and clinicopathological variables of 110 cases of HCC

Clinicopathologic Variables	n	miR-425 expression		*P*
		Low	High	
Gender				
Male	95	46	49	
Female	15	9	6	0.409
Age (years)				
≤ 60	85	42	43	
> 60	25	13	12	0.822
Liver cirrhosis				
Presence	89	45	44	
Absence	21	10	11	0.637
Liver function				
Child-Pugh A	101	51	50	
Child-Pugh B	9	4	5	0.731
Tumor size (cm)				
≤5	71	36	35	
> 5	39	19	20	0.844
Tumor nodule number				
Solitary	85	47	38	
Multiple(≥2)	25	8	17	**0.041**
Edmondson-Steiner grade				
I-II	46	21	25	
III-IV	64	34	30	0.444
Microvascular invasion				
Absence	58	35	23	
Presence	52	20	32	**0.022**
TNM stage				
Stage I	42	27	21	
Stage II	48	24	18	
Stage III	20	4	16	**0.028**
BCLC stage				
BCLC A	88	50	38	
BCLC B	22	5	17	**0.004**

We next evaluated the relationship between miR-425-5p expression and patient prognosis. Survival curves indicated that the 4-year overall survival (OS) and disease-free survival (DFS) of patients with high miR-425-5p expression were 26.8% and 19.7%, respectively, which were significantly lower than those of patients with low miR-425-5p expression (58.4% and 56.5%, respectively; *P* < 0.001; Figure 1C and 1D). The mean survival time in the high miR-425-5p group was significantly shorter than in the low miR-425-5p group (mean OS: 41.6 vs. 31.0 months; mean DFS: 36.0 vs. 24.4 months).

**Figure 2:**
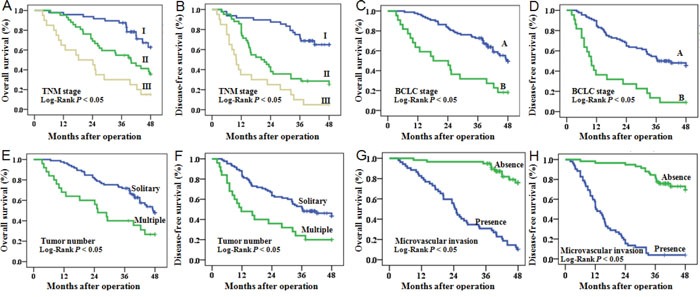
OS and DFS of HCC patients Patients were stratified into different subgroups according to pathological characteristics of HCC, including TNM stage **(A, B)**, BCLC stage **(C, D)**, tumor number **(E, F)**, and microvascular invasion **(G, H)**.

Univariate and multivariate survival analysis indicated that microvascular invasion (*P* < 0.001), TNM stage (*P* = 0.002), BCLC stage (*P* = 0.014), and miR-425-5p expression (*P* = 0.001) were independent risk factors for OS (Figure 2 and Supplementary Table 2). Univariate and multivariate survival analysis also confirmed that microvascular invasion (*P* < 0.001), BCLC stage (*P* = 0.042), and miR-425-5p expression (*P* = 0.002) were independent risk factors for DFS (Figure 2 and Supplementary Table 3).

### MiR-425-5p promotes invasion and migration of HCC cells *in vitro*

We first analyzed the expression of miR-425-5p in a panel of HCC cell lines compared to L02 normal liver cells (Supplementary Figure 1A). MiR-425-5p was over-expressed in all HCC cell lines, particularly in HCCLM3 cells, which had the highest metastatic potential. SMMC7721 and HCCLM3 cells were selected for further analysis. MiR-425-5p over- or under-expressing stable cell lines were constructed by infecting HCC cells with miR-425-5p- or anti-miR-425-5p-expressing lentiviral vectors. MiR-425-5p expression in these cell lines was quantified using qRT-PCR (Supplementary Figure 1B and 1C).

**Figure 3:**
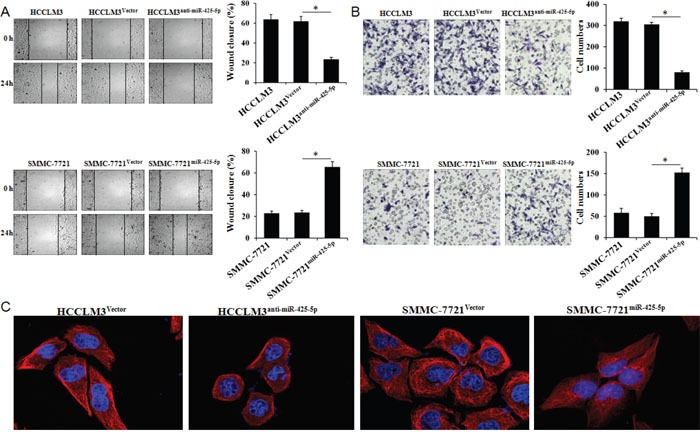
MiR-425-5p promotes HCC cell migration and invasion *in vitro* Wound healing **(A)** and transwell **(B)** assays were performed to analyze the effects of miR-425-5p on HCC cell migration and invasion. The percentage of wound closure and percentage of cells that migrated through the transwell membranes are shown. *, *P* < 0.05. **(C)** Representative immunofluorescence images showing miR-425-5p-induced changes in HCC cellular morphology. Cell nuclei were stained with DAPI (blue), and the cytoskeleton was stained with actin-tracker FITC (red). Original magnification ×400.

No significant differences were observed in cell proliferation, colony number, cell cycle distribution, or apoptosis ratio in response to miR-425-5p knockdown or over-expression (Supplementary Figure 2 and 3). However, wound healing assays showed that high levels of miR-425-5p were correlated with faster wound healing rates (Figure 3A). Similarly, transwell assays with Matrigel revealed that HCCLM3^anti-miR-425-5p^ cells displayed reduced ability to invade compared to control cells, whereas SMMC-7721^miR-425-5p^ cells migrated faster than control cells (Figure 3B).

Changes in cell morphology were observed by confocal fluorescence microscopy. Cells with relatively high miR-425-5p expression were elongated and displayed more filopodia-like protrusions (Figure 3C). Over-expression of miR-425-5p expression in Bel-7402 cells had no effect on cell proliferation, colony number, cell cycle distribution, or apoptosis, but enhanced the invasive ability of HCC cells (Supplementary Figure 4). These data indicated that miR-425-5p could enhance HCC migration and invasion.

### SCAI and PTEN are downstream targets of miR-425-5p

**Figure 4:**
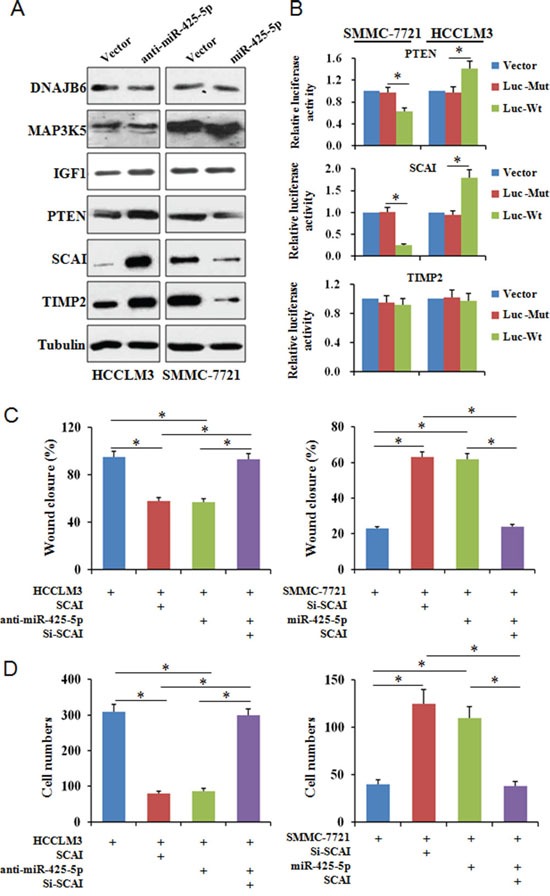
MiR-425-5p suppresses SCAI expression **(A)** Analysis of candidate target gene expression by western blotting. **(B)** Reporter plasmids with wild-type or mutant 3′ UTR sequences of PTEN, SCAI, and TIMP2 were transfected into HCCLM3 and SMMC-7721 cells infected with anti-miR-425-5p or miR-425-5p lentiviruses and relative luciferase activity analyzed. To investigate whether SCAI expression either interfered with or mimicked the function of miR-425-5p, HCC cells were infected with lentiviral vectors expressing SCAI siRNA or SCAI to inhibit or restore SCAI expression, respectively. Wound healing **(C)** and invasion **(D)** assays were performed using the above cells. * *P* < 0.01.

TargetScan, PicTar, miRanda, and PubMed were used to search and identify potential metastasis-related miR-425-5p target genes. Among the predicted targets, DNAJB6 (DnaJ heat shock protein family [Hsp40] member B6), MAP3K5 (mitogen-activated protein kinase kinase kinase 5), IGF1 (insulin like growth factor 1), PTEN (phosphatase and tensin homolog), SCAI (suppressor of cancer cell invasion), and TIMP2 (TIMP metallopeptidase inhibitor 2) were of particular interest. We analyzed the protein levels of these genes in response to miR-425-5p over-expression or knock-down. These results indicated that miR-425-5p could suppress PTEN, SCAI, and TIMP2 expression (Figure 4A).

We next investigated whether miR-425-5p could bind directly to the 3′ untranslated region (UTR) of PTEN, SCAI, or TIMP2. The wild-type or mutant 3′ UTR target sequences (Supplementary Figure 5A) were cloned into a luciferase reporter vector, pGL3, and then transfected into cells with the pRL-TK vector as a control. MiR-425-5p inhibited the luciferase activity of the wild-type 3′ UTR of PTEN and SCAI (Figure 4B). SCAI has two miR-425-5p binding sites. Our luciferase assays indicated that miR-425-5p could bind to both sites and suppress SCAI expression (Supplementary Figure 5B). We observed reduced SCAI expression in HCC tissue compared to ANLT, and miR-425-5p and SCAI expression were inversely correlated in HCC tissue (Supplementary Figure 6). These data indicate that miR-425-5p could inhibit PTEN and SCAI expression by directly targeting the 3′ UTRs of both genes.

### SCAI inhibits HCC cell invasion and migration *in vitro*

**Figure 5:**
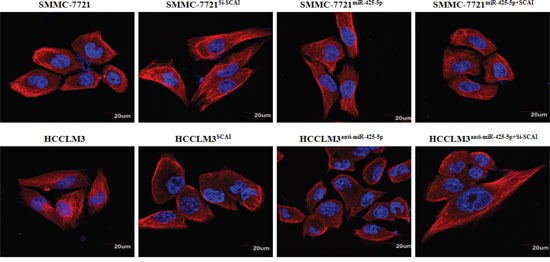
Representative immunofluorescence images of the cytoskeleton showing the cellular morphology in each group Cell nuclei were stained with DAPI (blue), while the cytoskeleton was stained with actin-tracker FITC (red). Original magnification ×400.

SCAI is a newly identified tumor suppressor that plays an important role in regulating cancer cell invasion [13]. However, the role of SCAI in HCC has not been investigated. Using a lentivirus expression system, we over-expressed or silenced SCAI expression in HCCLM3 and SMMC-7721 cells (Supplementary Figure 7). Wound healing and transwell invasion assays revealed that silencing SCAI expression enhanced the migration and invasion abilities of SMMC-7721 cells. In contrast, over-expression of SCAI inhibited HCCLM3 cell migration and invasion (Figure 4C and 4D, Supplementary Figure 8-10). Changes in cell morphology were also observed after ectopic expression of SCAI (Figure 5). These data indicated that SCAI could inhibit the invasion and migration of HCC cells *in vitro*, and confirmed that SCAI functions as a tumor suppressor in HCC.

### MiR-425-5p suppresses SCAI expression resulting in dysregulation of multiple signaling pathways

To examine whether miR-425-5p functioned through SCAI, we restored SCAI expression in HCCLM3^anti-miR-425-5p^ and SMMC-7721^miR-425-5p^ cells (Supplementary Figure 11). Wound healing and transwell assays revealed that silencing of SCAI mimicked the function of anti-miR-425-5p resulting in increased migration and invasion of HCCLM3 cells. Conversely, ectopic expression of SCAI blocked miR-425-5p-induced SMMC-7721 cell migration and invasion (Figure 4C and 4D, Supplementary Figure 8-10). Immunofluorescence analysis demonstrated that the morphological changes observed in HCCLM3^anti-miR-425-5p^ and SMMC-7721^miR-425-5p^ cells could be blocked by restoring SCAI expression (Figure 5).

**Figure 6:**
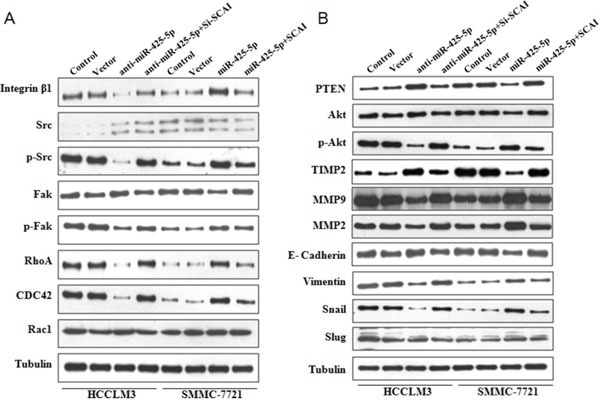
Western blot analysis of protein expression in HCC cells following ectopic expression or silencing of miR-425-5p, as well as ectopic expression or silencing of SCAI

SCAI was previously shown to suppress cancer cell invasion through transcriptional regulation of integrin β1 (ITGB1) [13]. Therefore, we evaluated the expression of genes downstream of ITGB1 by western blotting. MiR-425-5p induced an increase in ITGB1, p-Src (Tyr419), p-Fak (Tyr397), RhoA, and CDC42, but not Rac1 expression (Figure 6A and Supplementary Figure 12). The effects of miR-425-5p were mimicked or antagonized by SCA1 knock-down and ectopic expression, respectively (Figure 6A and Supplementary Figure 12). These data indicated that miR-425-5p could activate ITGB1- Fak/Src-RhoA/CDC42 signaling through suppression of SCAI expression.

**Figure 7:**
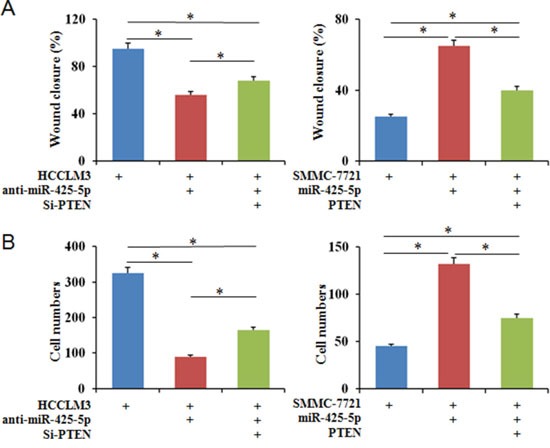
PTEN partially blocks the effects of miR-425-5p on the migration and invasion of HCC cells Wound healing **(A)** and transwell **(B)** assays were performed to analyze the effects of PTEN on miR-425-5p-induced HCC cell motility and invasion. The percentage of wound closure and percentage of cells that migrated through the transwell membranes are shown. * *P* < 0.05.

**Figure 8:**
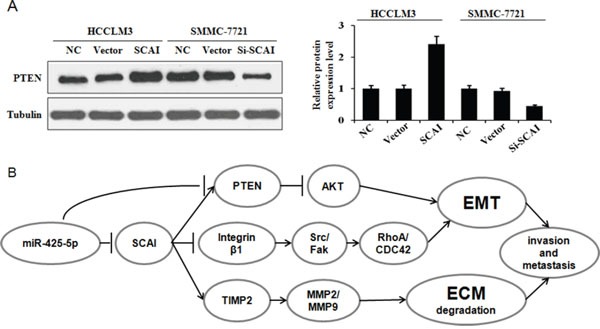
**(A)** SCAI stimulates PTEN expression Western blot analysis of PTEN expression in response to ectopic expression or silencing of SCAI. The relative expression of PTEN in these cells is. **(B)** MiR-425-5p promotes EMT and ECM degradation in HCC through suppression SCAI-mediated deregulation of the ITGB1-Fak/Src-RhoA/CDC42, PTEN-AKT, and TIMP2-MMP2/MMP9 signaling pathways.

Because PTEN is a downstream target of miR-425-5p, we investigated the role of PTEN in miR-425-5p-mediated HCC cell migration and invasion. MiR-425-5p function was partially rescued by PTEN (Figure 7). Interestingly, PTEN protein levels were increased in response to ectopic expression of SCAI in HCCLM3 cells. PTEN levels decreased after SCAI knockdown in SMMC-7721 cells (Figure 8A). The miR-425-5p- induced inhibition of PTEN expression could be reversed when SCAI expression was restored (Figure 6B and Supplementary Figure 12). These results suggested that PTEN was downstream of SCAI. The expression of AKT, which is a critical gene downstream of PTEN, was also examined. MiR-425-5p induced expression of p-AKT (Ser473), which was antagonized or mimicked by restoring SCAI expression (Figure 6B and Supplementary Figure 12). These data indicated that miR-425-5p could stimulate PTEN-AKT signaling by suppressing SCAI expression.

Although the TIMP2 is not a direct target of miR-425-5p, miR-425-5p can inhibit TIMP2 expression. Importantly, miR-425-5p-mediated inhibition of TIMP2 expression could be reversed by restoring SCAI expression (Figure 6B and Supplementary Figure 12), suggesting that miR-425-5p inhibited TIMP2 expression through SCAI. We also analyzed the expression of MMP2 and MMP9, which are downstream of TIMP2. Over-expression of miR-425-5p resulted in elevated expression of MMP2 and MMP9, which was inhibited by miR-425-5p knockdown. These effects were antagonized or mimicked by restoring SCAI expression (Figure 6B and Supplementary Figure 12). These data demonstrated that miR-425-5p could inhibit SCAI and activate TIMP2-MMP2/MMP9 signaling. Thus, miR-425-5p exerts its function through SCAI-mediated dysregulation of the ITGB1-Fak/Src-RhoA/CDC42, PTEN-AKT, and TIMP2-MMP2/MMP9 signaling pathways (Figure 8B).

### MiR-425-5p promotes EMT in HCC cells

Immunofluorescence analysis revealed morphological changes (i.e. mesenchymal-like morphology) in HCC cells after ectopic expression of miR-425-5p (Figure 3C and Figure 5), suggesting that miR-425-5p could regulate EMT. To test this hypothesis, we analyzed the expression of EMT-related markers including E-cadherin, vimentin, Snail, and Slug. Inhibition miR-425-5p in HCCLM3 cells resulted in a decrease in vimentin and an increase in E-cadherin expression. In contrast, over-expression of miR-425-5p in SMMC-7721 cells resulted in an increase in vimentin and a decrease in E-cadherin expression. Furthermore, miR-425-5p promoted the expression of Snail and Slug. Importantly, all of the miR-425-5p-mediated effects were mimicked or antagonized by SCAI silencing and ectopic expression, respectively (Figure 6B and Supplementary Figure 12). Immunohistochemical analysis also demonstrated reduced E-cadherin and increased vimentin, Snail, and Slug expression in HCC tissues with high miR-425-5p expression (Supplementary Figure 13). These data indicated that miR-425-5p promoted EMT through SCAI-mediated activation of downstream Snail/Slug signaling.

### MiR-425-5p promotes HCC metastasis *in vivo*

**Figure 9:**
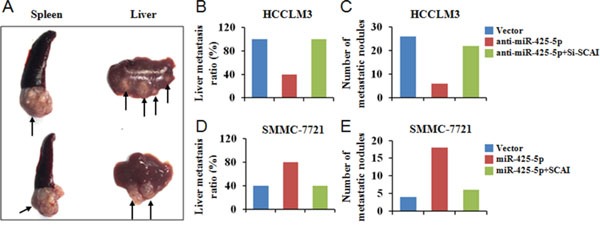
MiR-425-5p promotes metastasis *in vivo* **(A)** Representative image of the tumor nodules observed at the primary (spleen) and metastatic (liver) sites. The black arrows indicate the locations of the primary tumor and metastatic nodules. **(B, D)** The number of mice with intrahepatic metastatic nodules. **(C, E)** The total number of intrahepatic metastatic nodules in each group.

We developed a mouse model of HCC metastasis in order to evaluate the functions of miR-425-5p *in vivo*. HCC cells were injected into the spleens of nude mice and the spleens and livers harvested after 6 weeks. All mice developed local tumors in the spleen (Figure 9A). Intrahepatic metastatic nodules were detected in all of the mice in the HCCLM3^Vector^ group, but in only four mice in the HCCLM3^anti-miR-425-5p^ group (Figure 9B). The total number of metastatic nodules in the liver decreased by 76.9% in the HCCLM3^anti-miR-425-5p^ compared to the control group (Figure 9C). Furthermore, intrahepatic metastatic nodules were detected in four mice in the SMMC-7721^Vector^ group, but in eight mice in the SMMC-7721^−miR-425-5p^ group (Figure 9D). The total number of metastatic nodules in the liver was approximately 4.5-fold higher in the SMMC-7721^miR-425-5p^ group compared to the control group (Figure 9E). MiR-425-5p-induced intrahepatic metastasis was antagonized by ectopic expression of SCAI (Figure 9B-9E). Additionally, immunohistochemistry demonstrated that miR-425-5p could facilitate EMT by regulating the expression of EMT-related genes (Supplementary Figure 14). These data indicated that miR-425-5p could enhance the migration and invasion of HCC cells *in vivo*.

## DISCUSSION

Hepatectomy, which is associated with a 5-year survival rate greater than 30%, is one of the best choices for HCC treatment. However, the rates of recurrence and metastasis after hepatectomy are high, which is a major obstacle to improving the long-term survival of HCC patients [14]. A better understanding of the molecular events underlying HCC metastasis is therefore critical. Although miRNAs have important roles in metastasis, the pathological relevance and significance of the majority of miRNAs in HCC remain unclear.

We found that miR-425-5p expression was elevated in tumor tissue from HCC patients and was correlated with poor clinicopathological characteristics and shorter long-term survival. Additionally, miR-425-5p expression was an independent risk factor for OS and DFS. Aberrant expression of miR-425 has been observed in multiple tumors. For example, a recent study found that miR-425-5p expression predicted the response to sorafenib therapy in HCC patients [15]. MiR-425-5p expression was also elevated in cervical cancer, and high serum miR-425-5p levels were associated with poor survival [16]. These data suggest that miR-425-5p may serve as a prognostic biomarker for various cancers.

Previous studies have shown that miR-425-5p plays important roles in cancer development. Upregulation of miR-425 inhibited PTEN expression, promoted cell proliferation, and protected cells from cisplatin-induced apoptosis in gastric cancer [17]. MiR-425-5p was also shown to promote invasion and metastasis of gastric cancer cells *in vitro* and *in vivo*, although the underlying mechanisms have not been described [18]. MiR-425 promoted cell proliferation and metastasis by targeting SMAD2 in esophageal squamous cell carcinoma [19]. We found that miR-425-5p could promote invasion and migration of HCC cells both *in vitro* and *in vivo*. However, miR-425-5p had no effect on proliferation and apoptosis in HCC cells. These data suggest that miR-425-5p has a critical role in cancer progression.

We investigated the mechanisms underlying miR-425-5p function in HCC progression. SCAI and PTEN were confirmed to be direct targets of miR-425-5p. SCAI is a highly conserved protein that regulates invasive cell migration. SCAI localizes to the nucleus where it binds and inhibits the myocardin-related transcription factor MAL by forming a ternary complex with serum response factor, and inhibits invasive cell migration through regulating ITGB1 transcription [13]. The myocardin-related transcription factor (MRTF) complex is an important regulator of fibrosis, tumor cell invasion, and metastasis [20]. As a negative regulator of MRTF, SCAI can regulate EMT and renal fibrosis [21]. SCAI expression was diminished in human breast cancer cells, and it was shown to regulate cancer cell invasion through an interaction with the SWI/SNF complex [22]. Here, we demonstrated that SCAI expression was reduced in HCC patient tissue specimens. Using a loss- and gain-of-function approach, we confirmed that SCAI inhibited HCC invasion and metastasis *in vitro*. Furthermore, SCAI knockdown mimicked, whereas SCAI over-expression antagonized, the functions of miR-425-5p, indicating SCAI is the primary downstream target of miR-425-5p in HCC.

Integrins are heterodimeric transmembrane receptors. ITGB1 forms a heterodimeric complex with various integrin subunits and regulates cell focal adhesion and tumor metastasis [23]. Masumoto et al. reported that a monoclonal antibody against ITGB1 blocked HCC cell invasion [24]. FAK has a key role in integrin signaling. When extracellular ligands bind to integrins, FAK is recruited to the C-terminal domains of integrins and is auto-phosphorylated (Tyr397), which creates a binding site for the Src-homology 2 (SH2) domain of Src [23]. The FAK-Src complex activates Rho GTPase family members (e.g. Cdc42, RhoA, and Rac1) [25]. Cdc42 stimulates filopodia formation, RhoA induces the formation of stress fibers, and Rac1 induces lamellipodia formation, all of which play critical roles in cancer cell invasion and metastasis [26]. We demonstrated that miR-425-5p promoted the expression of ITGB1, p-FAK, p-Src, RhoA, and CDC42, and that ectopic expression of SCAI could reverse the effects of miR-425-5p.

PTEN is one of the most frequently mutated tumor suppressors in human cancers [27]. It can suppress cell motility/migration through a variety of pathways. PI3K/AKT is one important target of PTEN [28]. In this study, we found that PTEN was a direct target of miR-425-5p. However, the function of miR-425-5p could only be partially rescued by PTEN over-expression. Interestingly, SCAI increased PTEN expression, and miR-425-5p-induced inhibition of PTEN expression was antagonized by SCAI, suggesting that PTEN is a downstream target of SCAI. Additional studies are required to elucidate the mechanism by which SCAI activates PTEN. MiR-425-5p induced up-regulation of p-AKT (Ser473), which could be antagonized or mimicked by restoring SCAI expression. These data indicated that miR-425-5p could activate the PTEN-AKT signaling pathway through suppressing SCAI expression. Thus, miR-425-5p could suppress PTEN expression through a SCAI-dependent or -independent pathway, and promote AKT phosphorylation resulting in increased cell migration and metastasis.

Degradation of the basement membrane and the extracellular matrix (ECM) by zinc-dependent matrix metalloproteinases (MMPs) is a prerequisite for tumour cell invasion [29]. Imbalances in secreted MMPs and the tissue inhibitors of MMPs (TIMPs) have been linked to the invasive behavior of tumor cells [30]. We found that TIMP2, which is not a direct target of miR-425-5p, could be inhibited by miR-425-5p. Ectopic expression of miR-425-5p increased MMP2 and MMP9 levels, while miR-425-5p knockdown decrease MMP2 and MMP9 expression. Additionally, miR-425-5p-induced changes in TIMP2, MMP2, and MMP9 expression could be antagonized by SCAI expression, suggesting that miR-425-5p stimulated TIMP2-MMP2/MMP9 signaling by suppressing SCAI expression.

EMT is essential for the dissemination of malignant cells to adjacent tissues and distant sites [31]. EMT-induced changes in epithelial cell plasticity are evidenced by the loss of epithelial markers such as E-cadherin, and an increase in the expression of mesenchymal proteins including vimentin and the EMT-associated transcription factors Snail and Slug [32]. We found that elevated miR-425-5p expression promotes EMT, whereas miR-425-5p knockdown resulted in the opposite effect. The EMT phenotype was reversed by ectopic expression of SCAI. Thus, miR-425-5p promotes EMT and metastasis through suppression of SCAI.

In conclusion, miR-425-5p is over-expressed in HCC and is correlated with poor patient prognosis. MiR-425-5p promotes HCC metastasis by suppressing SCAI- mediated dysregulation of the ITGB1-Fak/Src-RhoA/CDC42, PTEN-AKT, and TIMP2-MMP2/MMP9 signaling pathways. MiR-425-5p may therefore be a prognostic biomarker and a novel therapeutic target for HCC.

## MATERIALS AND METHODS

### Patients and tissue specimens

Paired HCC tissue and ANLT samples were collected from 110 patients who underwent liver resection between December 2011 and December 2013 in the Department of Hepatobiliary Cancer at Tianjin Medical University Cancer Institute and Hospital. The samples were snap-frozen in liquid nitrogen and stored at -80°C prior to RNA extraction or the generation of formalin-fixed, paraffin embedded tissue sections for immunohistochemistry.

Conventional clinicopathological data including age, gender, presence of liver cirrhosis, Edmondson-Steiner grade, tumor size, number of tumor nodes, microvascular invasion, Child-Pugh classification, TNM stage, and BCLC stage were collected. Microvascular invasion was defined as the presence of malignant cells in peritumoral vessels, which was only determined after a careful histological assessment of the entire surgical specimen [11]. The clinicopathological features of the patients are shown in Supplementary Table 1. All human specimens were obtained with informed consent and approved by the Ethics Committee of Tianjin Medical University Cancer Institute and Hospital.

### Follow-up and prognostic studies

Follow-up was performed for all patients. The follow-up period was defined as the time from the date of surgery to the date of either death or the last follow-up. Deaths from other causes were treated as censored cases. Recurrence and metastasis were diagnosed based on serial alpha-fetoprotein levels, ultrasonography, or computed tomography/magnetic resonance imaging. DFS was defined as the length of time that a patient survived following hepatectomy without any signs of HCC.

### Quantitative RT-PCR

Total RNA was reverse-transcribed into complementary DNA using the PrimeScript RT reagent Kit (TaKaRa, Dalian, China) and a gene-specific reverse transcription primer (Applied Biosystems, Foster City, CA, USA). We performed qRT-PCR using TaqMan microRNA assays (Applied Biosystems) to quantify the relative expression of miR-425-5p in tumor compared to ANLT, and in HCC cell lines. Expression was normalized to that of the endogenous U6 small nuclear RNA. The miR-425-5p primers were the following: forward: TGCGGAATGACACGAT- CACTCCCG; reverse: CCAGTGCAGGGTCCGAGGT. Relative fold changes in expression were calculated using the comparative Ct (2^−ΔΔCt^) method.

### Cell lines and cell culture

All experiments were performed using normal liver cell lines (L02, primary human hepatocytes) and five HCC cell lines (Bel-7402, SMMC7721, MHCC97-L, MHCC97-H, HCCLM3). The L02, SMMC7721, and Bel-7402 cells were purchased from the Cell Bank of Typical Culture Preservation Committee of Chinese Academy of Science (Shanghai, China). The MHCC97-L, MHCC97-H, and HCCLM3 cell lines were provided by the Liver Cancer Institute of Fudan University (Shanghai, China). All cell lines were cultured in Dulbecco's Modified Eagle's Medium supplemented with 10% fetal bovine serum and antibiotics at 37°C with 5% CO2.

### Vector construction

The miR-425-5p fragment was amplified from genomic DNA and inserted into the lentivirus expression vector pGCL (GenePharma, Shanghai, China). The over-expression vector was constructed by inserting the CDS sequences into the pEX-2 vector (GeneChem, Shanghai, China). The wild-type and mutant 3′ UTR sequences of SCAI, PTEN, and TIMP2 were amplified from human liver genomic DNA and then cloned into the downstream region of the firefly luciferase cassette in the pGL3 vector.

### Cell proliferation and colony formation assays

Cell viability was assessed using the MTT Cell Proliferation and Cytotoxicity Assay Kit (Beyotime, Beijing, China) according to the manufacturer's instructions. The optical density values were measured at 490 nm using an ELISA reader (Bio-Rad 680, Hercules, CA, USA). For colony formation assays, 500 cells were seeded into 35 mm dishes (Corning, Corning, NY, USA) and cultured for 2 weeks at 37°C. The number of colonies per dish was counted after staining the cells with crystal violet. Only positive colonies (diameter > 40 μm) were counted and analyzed. All experiments were performed in triplicate.

### Flow cytometry

Cell cycle analysis was performed with flow cytometry using a propidium iodide cell cycle detection kit (Beyotime, Beijing, China) and a flow cytometer (BD, Franklin Lakes, NJ, USA). Apoptosis was analyzed using the ApoScreen Annexin V Apoptosis Kit (SouthernBiotech, Birmingham, AL, USA) according to the manufacturer's instructions.

### *In vitro* wound healing assays

Cells were seeded onto 35 mm dishes (Corning) that were coated with fibronectin. After the cells reached 100% confluence, they were pre-incubated with mitomycin (Sigma, St. Louis, MO, USA; 10 μg/mL) for 1 h at 37°C to inhibit cell proliferation, which could confound the analysis of cell migration. Wound healing assays were performed by scratching confluent cell monolayers with a sterile pipette tip. The media was then exchanged and the cells were cultured for 24 h. The percentage of wound closure was calculated for five randomly chosen fields.

### Invasion assays

For invasion assays, 1 × 10^5^ cells in serum-free media supplemented with 0.1% bovine serum albumin were placed into the upper chamber of the insert with Matrigel (BD Biosciences, Franklin Lakes, NJ, USA). After 24 h of incubation at 37°C, we removed the cells that remained in the upper chamber or membrane. We counted the number of cells adhering to the lower membrane of the inserts after staining with a solution containing 0.1% crystal violet (Beyotime, Beijing, China) and 20% methanol. The invasion assays for each experimental group were performed in triplicate, and three random fields in each replicate were selected for quantification.

### Western blotting

Total protein was extracted and separated by SDS-PAGE and then transferred onto PVDF membrane (Millipore, Bedford, MA, USA). All primary antibodies were purchased from Santa Cruz Biotechnology (Santa Cruz, CA, USA). β-tubulin (Sigma) was used as a loading control.

### Luciferase reporter assays

The wild-type or mutant of 3′ UTR sequences were inserted into the pGL3 vector (GeneChem, Shanghai, China). After infection with the lentivirus or negative control, the cells were seeded into 96-well plates. We then co-transfected the cells with 50 ng pGL3 and 10 ng of the pRL-TK using Lipofectamine LTX (Invitrogen, Carlsbad, CA, USA). The cells were harvested after 24 h according to the manufacturer's protocol (Promega, Madison, WI, USA) and firefly and Renilla luciferase activity detected using the Dual-luciferase Reporter Assay System (Promega) and a Victor X Multilabel Plate Reader (Perkin-Elmer, Boston, MA, USA).

### HCC mouse model

The HCC mouse model was constructed as described previously [12]. Briefly, 5 × 10^6^ HCC cells for each experimental group were suspended in 40 μL of serum-free RPMI 1640/Matrigel (1:1) per mouse. Each group contained 10 female BALB/c-nu/nu nude mice that were 5–6 weeks of age. The mice were anesthetized and the cells injected into the upper pole of the spleen using a microsyringe. The mice were sacrificed after 6 weeks, the spleens and livers harvested, the tissue fixed in phosphate-buffered neutral formalin for histological analysis. The livers were dissected into small pieces in order to count the total number of visible intrahepatic metastasis nodules. Mice were manipulated and housed at the Animal Institute according to the protocols approved by the Medical Experimental Animal Care Commission.

### Statistical analysis

Statistical analysis was performed using the SPSS software (version 13.0, Chicago, IL, USA). Differences between two groups were analyzed using Student's t tests (comparison between two groups) or by one-way analysis of variance (ANOVA; comparison between more than two groups). The association between miR-425-5p expression and various clinicopathological variables were analyzed using Spearman's correlation tests. Survival analysis was performed using the Kaplan-Meier method and log-rank tests. Cox proportional hazard regression models were used to identify factors that were independently associated with OS and DFS. A P value < 0.05 (two-tailed) was considered statistically significant.

## SUPPLEMENTARY MATERIALS FIGURES AND TABLES


