# Supplementary material for: MiR-425-5p promotes invasion and metastasis of hepatocellular carcinoma cells through SCAI-mediated dysregulation of multiple signaling pathways
Source: Oncotarget. 2017 Mar 7;8(19):31745–57. doi: 10.18632/oncotarget.15958 (PMC5458244; doi:10.18632/oncotarget.15958)
Supplement: Supplementary file 1 [file oncotarget-08-31745-s001.pdf]

MiR-425-5p promotes invasion and metastasis of hepatocellular carcinoma cells through SCAI-mediated dysregulation of multiple signaling pathways

SUPPLEMENTARY FIGURES AND TABLES

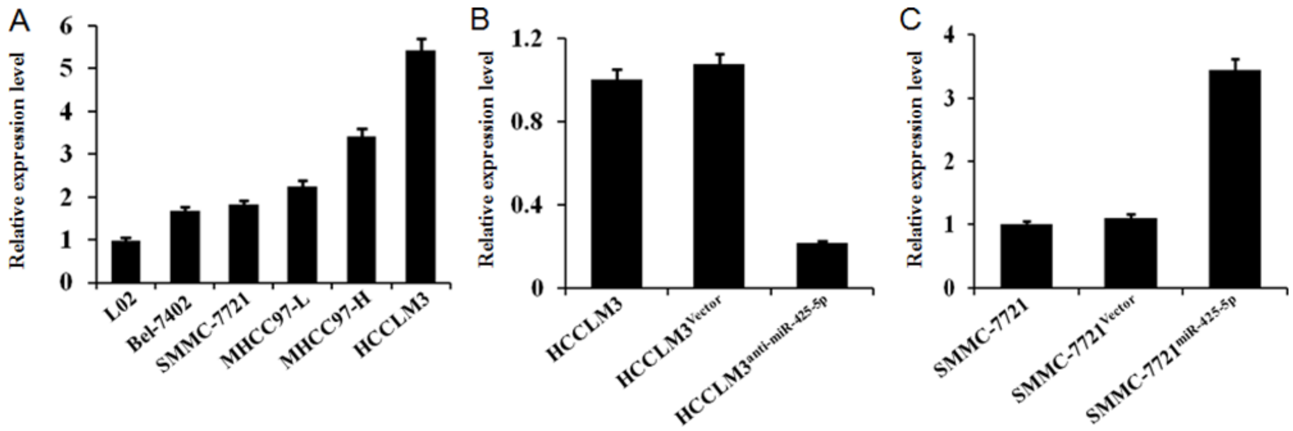

Supplementary Figure 1: (A) The expression of miR-425-5p in normal liver cell line (L02) and five HCC cell lines (Bel-7402, SMMC7721, MHCC97-L, MHCC97-H and HCCLM3) was analyzed by qRT-PCR. The data was normalized against the expression level of miR-425-5p in L02 cells. The expression of miR-425-5p was confirmed by qRT-PCR after infected with anti-miR-425-5p **(B)** or miR-425-5p lentivirus **(C)**. The miR-425-5p expression in HCCLM3 or SMMC7721 cells was set as 1.

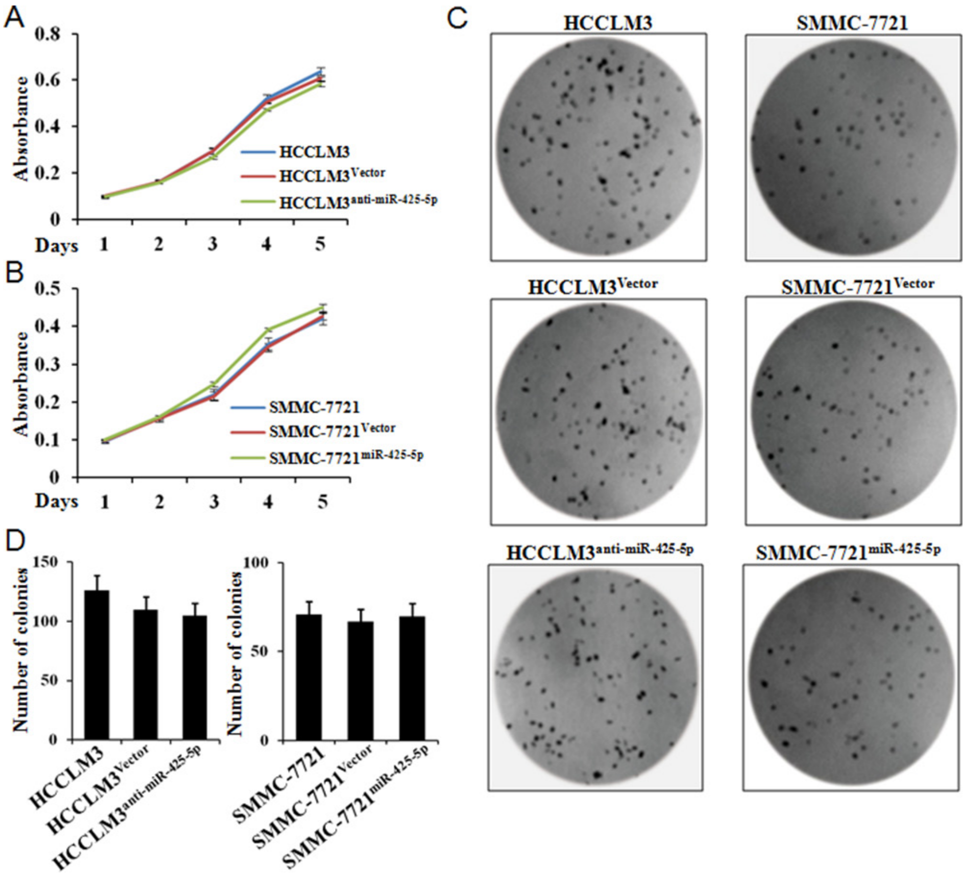

Supplementary Figure 2: miR-425-5p has no effect on colony formation and cell proliferation of HCC cells. HCCLM3 infected with anti-miR-425-5p lentivirus or vector control and SMMC7721 infected with miR-425-5p or vector control. The growth curve **(A, B)** and colony formation assay **(C)** of these cells were analyzed. **(D)** The numbers of colonies of each group were counted.

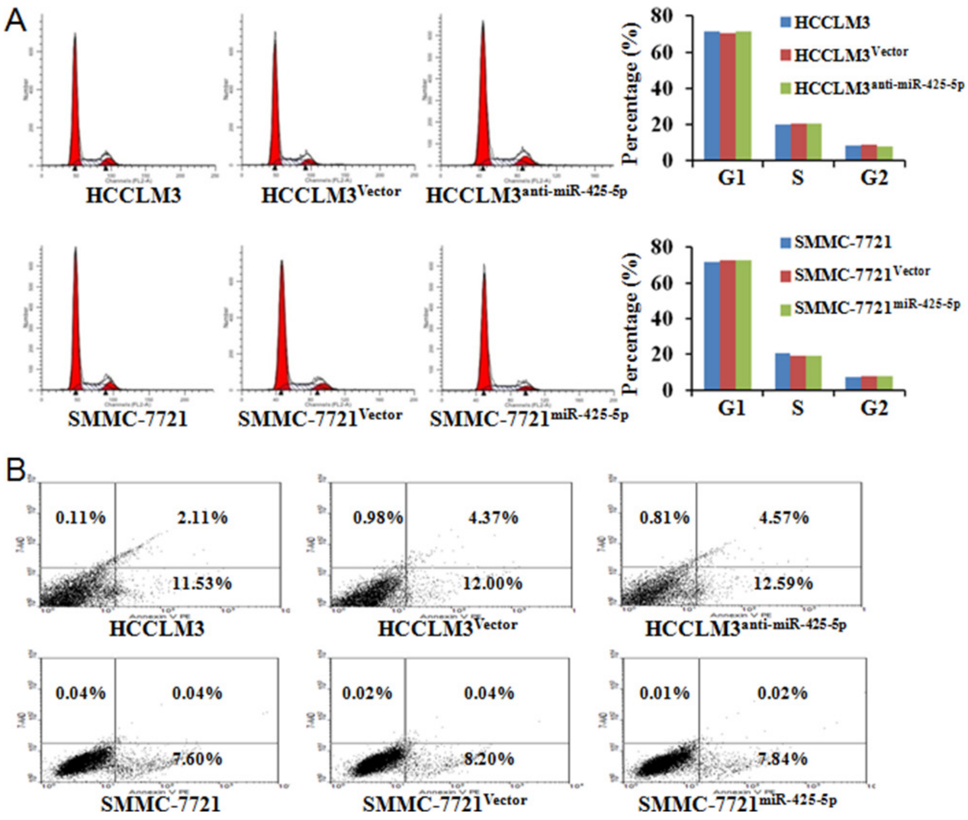

Supplementary Figure 3: miR-425-5p has no effect on cell cycle distribution and apoptosis of HCC cells. HCCLM3 infected with anti-miR-425-5p lentivirus or vector control and SMMC7721 infected with miR-425-5p or vector control. The cell distribution **(A)** and apoptosis **(B)** of these cells were analyzed by flow cytometry assay.

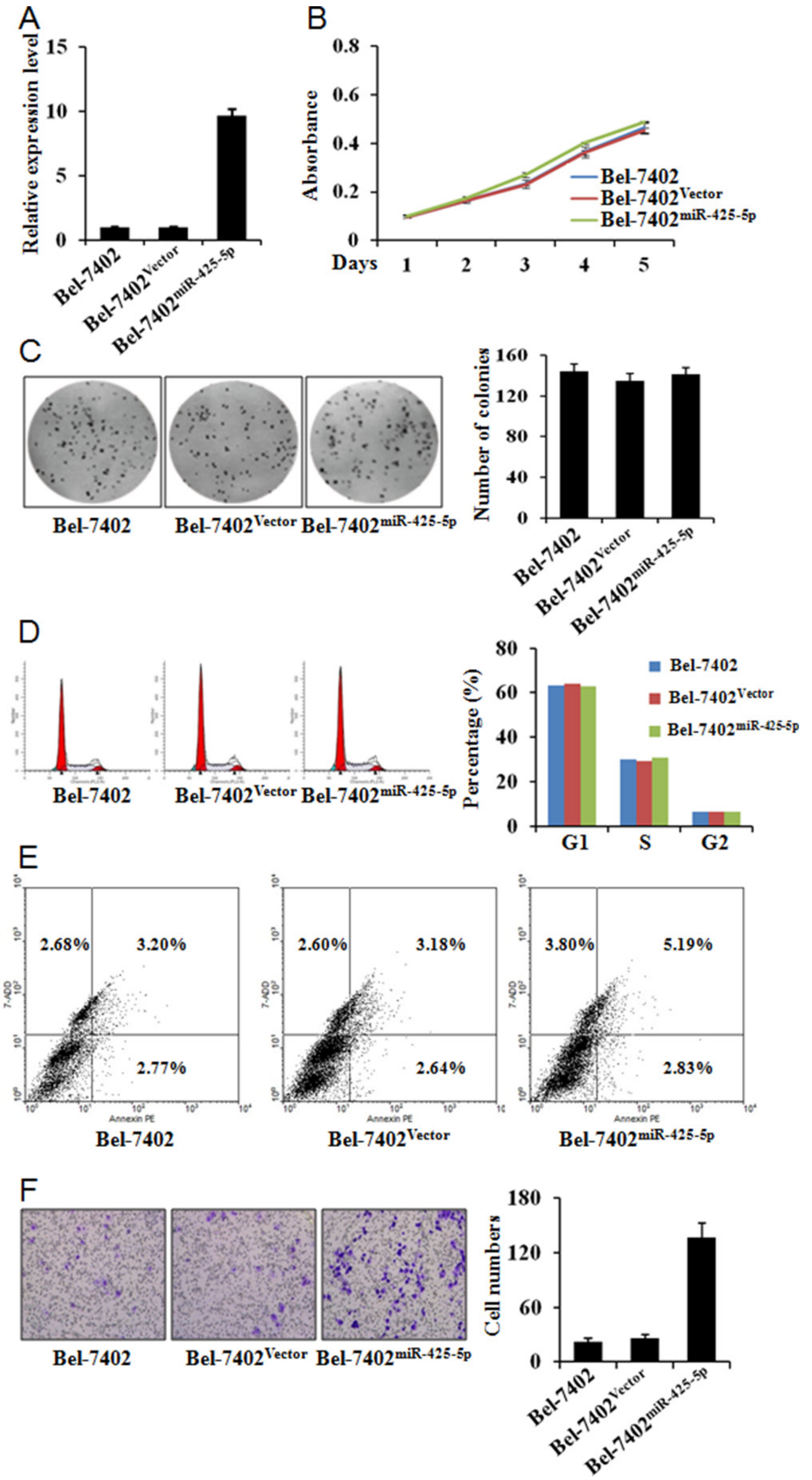

Supplementary Figure 4: miR-425-5p promotes invasion but not others in Bel-7402 cells. (A), Bel-7402 cells were infected with miR-425-5p lentivirus vector control. The expression of miR-425-5p was analyzed by qRT-PCR. The data was normalized against the expression level of miR-425-5p in Bel-7402 cells. The MTT assays **(B)**, colony formation assay **(C)**, flow cytometry analysis **(D, E)** and transwell assay **(F)** were performed. The data of colony formation and transwell assay was calculated and compared in the corresponding diagrams.

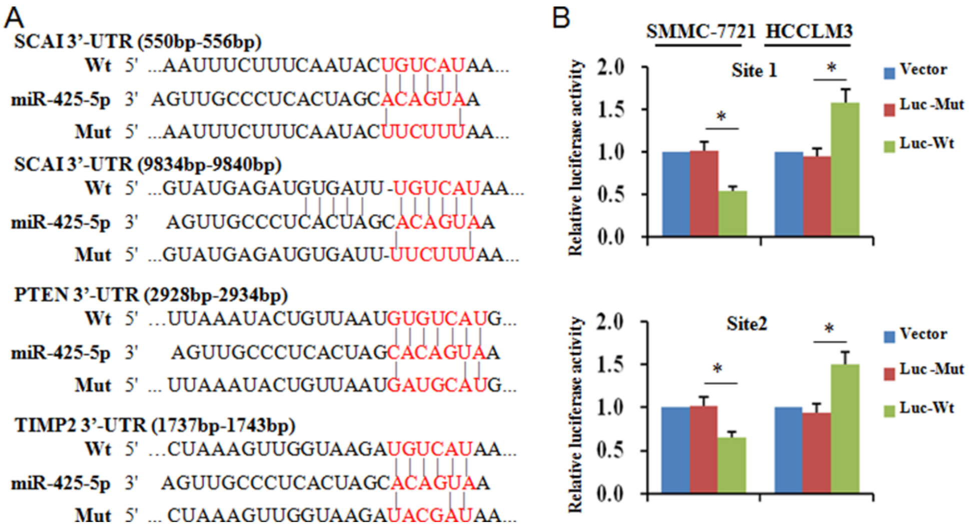

Supplementary Figure 5: SCAI is a direct downstream target for miR-425-5p in HCC cells. **(A)** The sequences of predicted miR-425-5p binding sites within the 3'-UTR of SCAI, PTEN and TIMP2, the wild-type or mutant binding site are shown. **(B)** The reporter plasmids with wild-type or mutant binding the 3'-UTR of SCAI site 1 or site 2 were transfected into HCCLM3 and SMMC-7721 cells infected with anti-miR-425-5p or miR-425-5p lentivirus. Relative luciferase activity was analyzed. * $P < 0.01$.

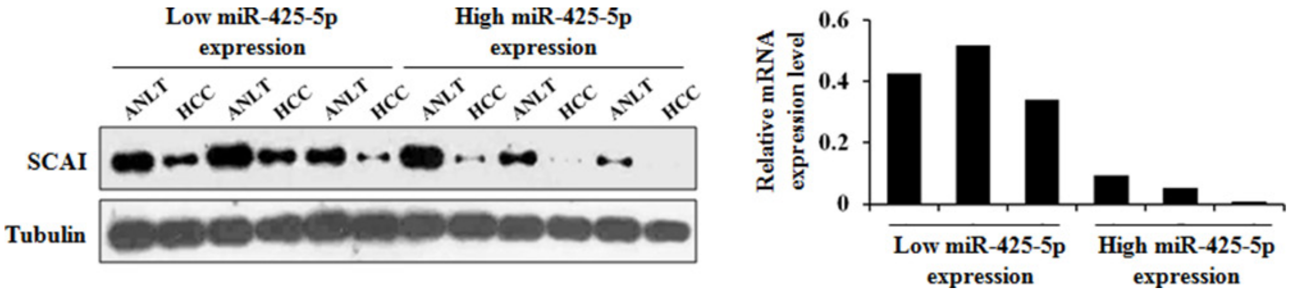

Supplementary Figure 6: The expression of SCAI protein in pairs of samples of HCC was tested with western blot.

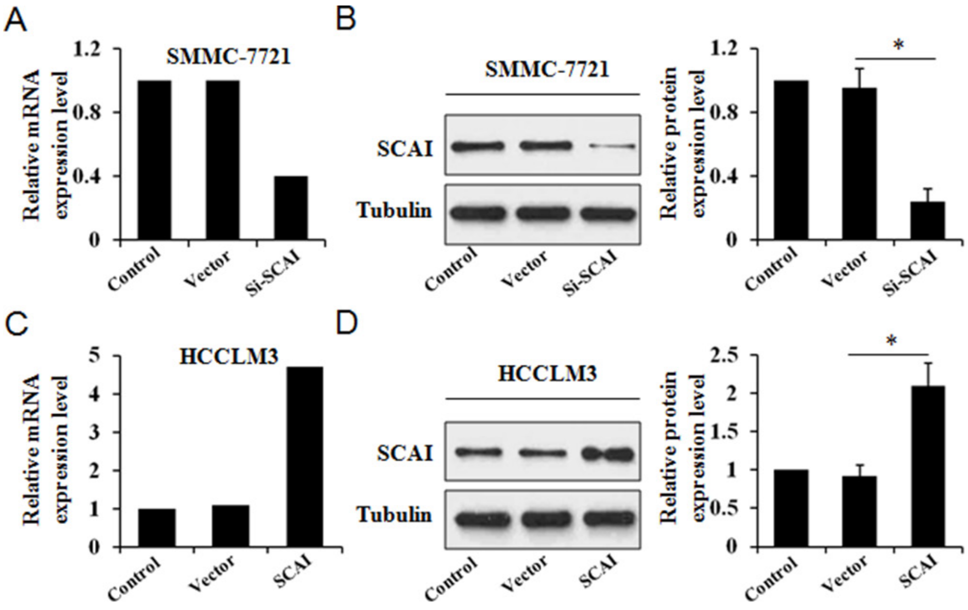

Supplementary Figure 7: The SCAI mRNA and protein expression levels were detected by qRT-PCR and western blot after SMMC-7721 (A and B) and HCCLM3 cells (C and D) infected with SCAI inhibition lentivirus or SCAI overexpression lentivirus. The data were normalized to the expression level of SCAI in untreated HCC cell. * $P < 0.01$.

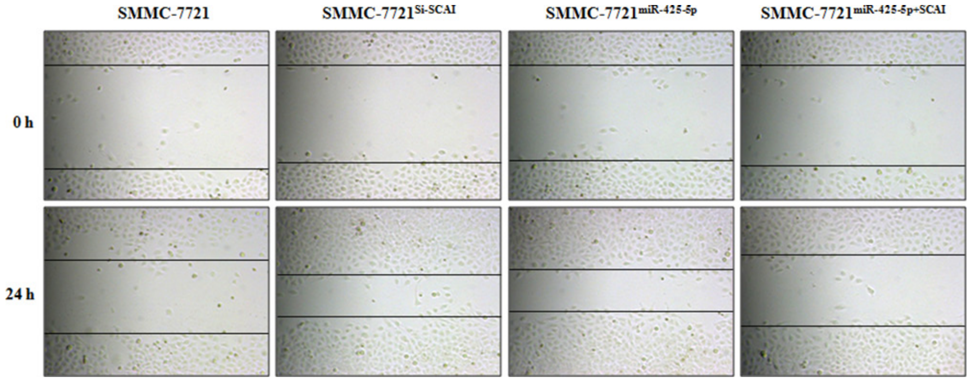

Supplementary Figure 8: The wound healing assay was performed to analyze the effect of miR-425-5p and SCAI on the motility of SMMC-7721 cell lines.

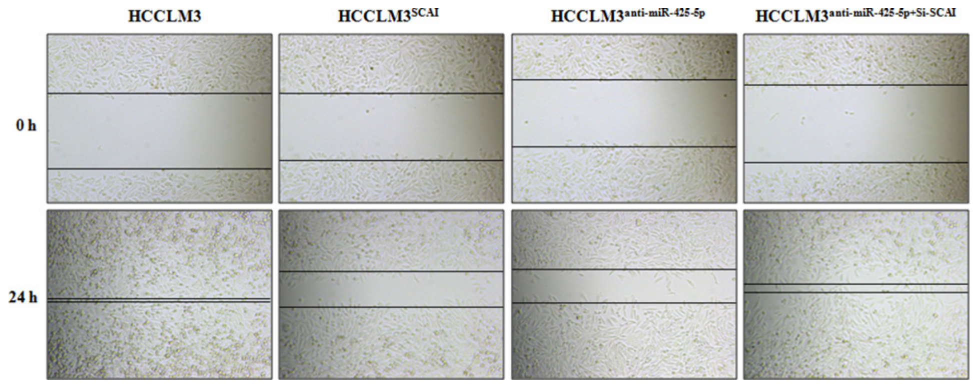

Supplementary Figure 9: The wound healing assay was performed to analyze the effect of miR-425-5p and SCAI on the motility of HCCLM3 cell lines.

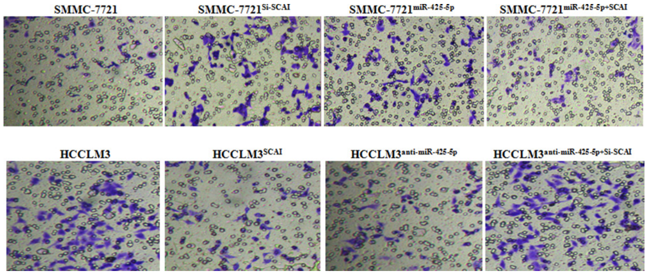

Supplementary Figure 10: The transwell assay were performed to analyze the effect of miR-425-5p and SCAI on the invasion of HCC cell lines.

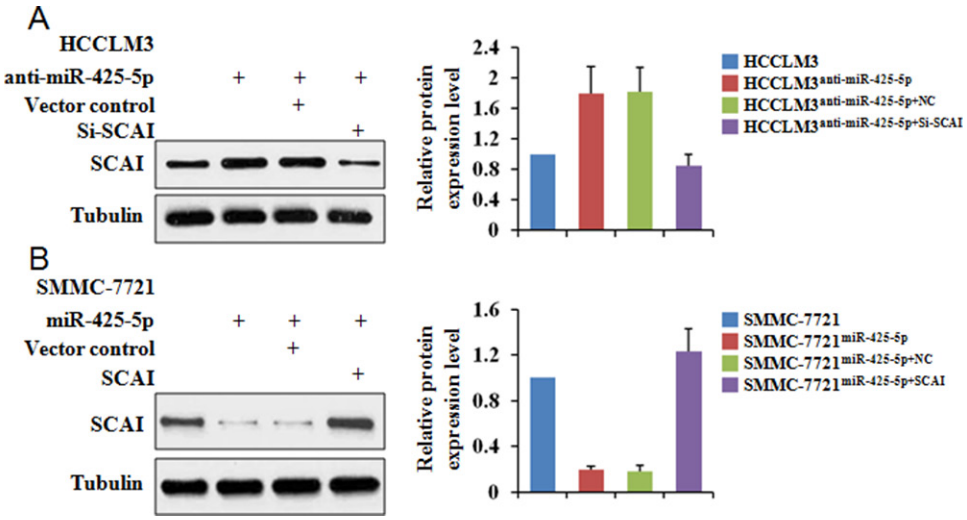

Supplementary Figure 11: The SCAI protein expression levels were detected by western blot after HCCLM3 (A) and SMMC-7721 cells (B) infected with different lentivirus. The data were normalized to the expression level of SCAI in untreated HCC cell.

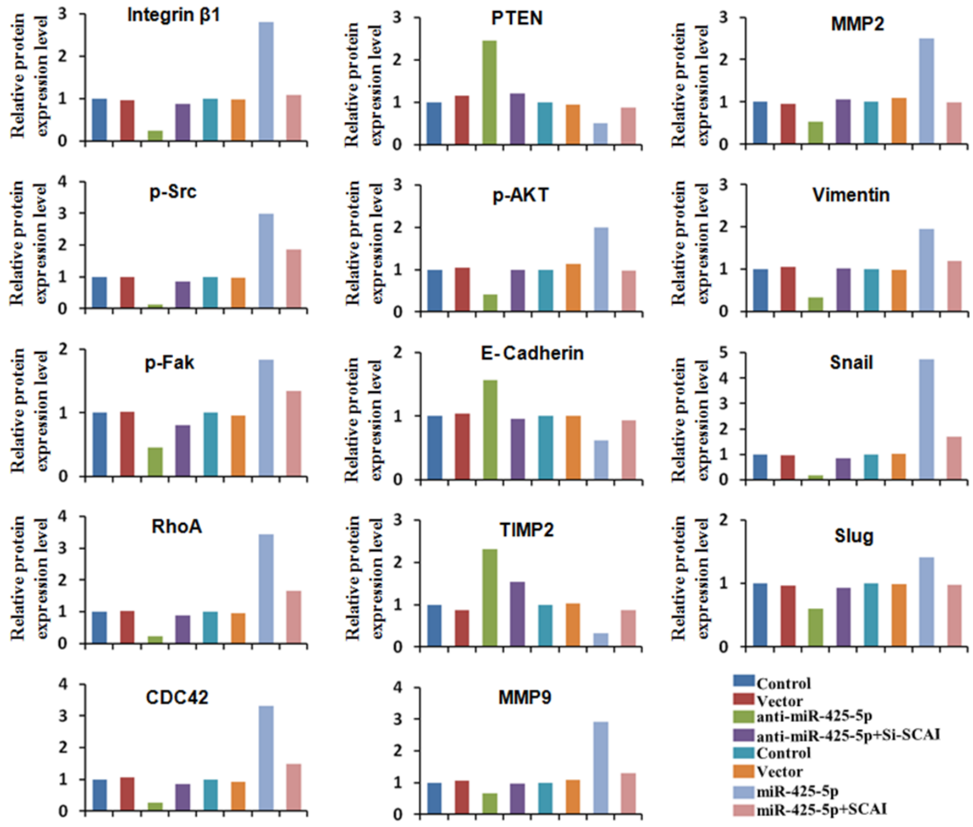

Supplementary Figure 12: Diagramsof protein expressions in different HCC cells treated with ectopic expression or silencing of miR-425-5p, as well as ectopic expression or silencing of SCAI. The data were normalized to the protein expression level in untreated HCC cell.

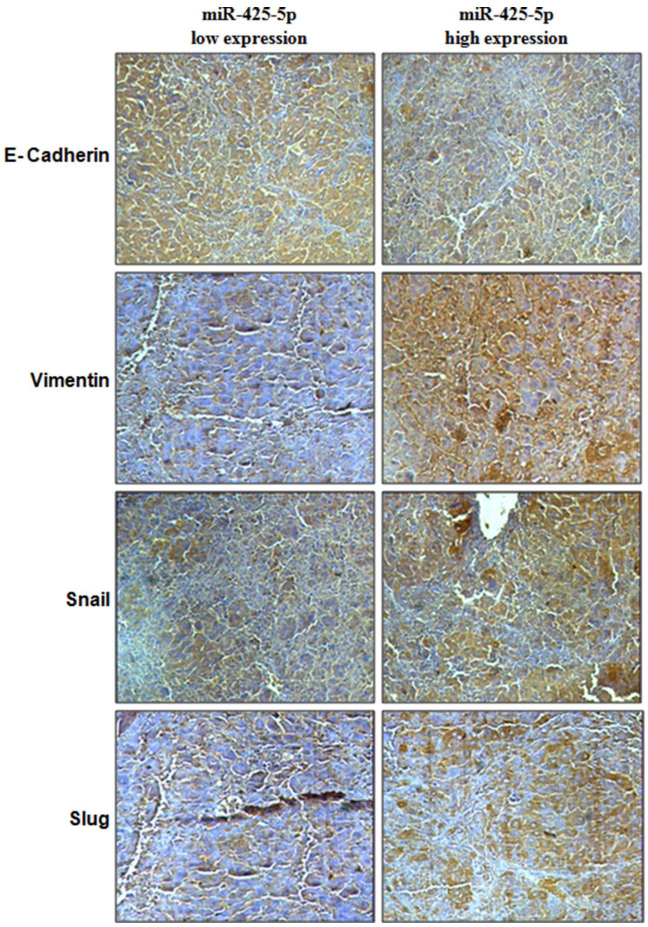

Supplementary Figure 13: The representative immunohistochemistry pictures showed the protein expression of EMT related gene in HCC tissue with high or low miR-425-5p expression.

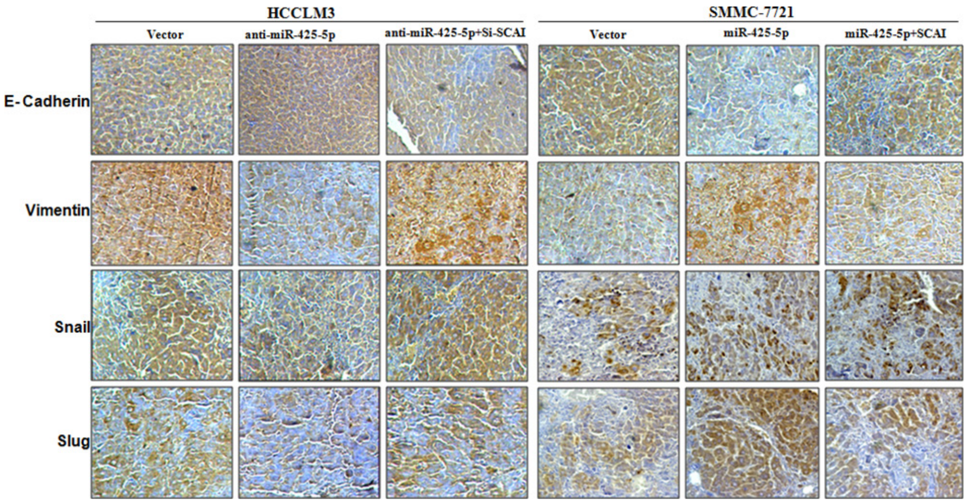

Supplementary Figure 14: The representative immunohistochemistry pictures showed the protein expression of EMT related gene in intrahepatic metastasis tumor tissues.

Supplementary Table 1: Clinicopathologcal characteristics of the 110 patients

Variables	N (%)
Gender	
Male	95 (86.4%)
Female	15 (13.6%)
Age (years)	50 (20-75)
≤ 60	85 (77.3%)
> 60	25 (22.7%)
Liver cirrhosis	
Presence	89 (80.9%)
Absence	21 (19.1%)
Liver function	
Child-Pugh A	101 (91.8%)
Child-Pugh B	9 (8.2%)
Tumor size (cm)	
≤5	71 (64.5%)
> 5	39 (35.5%)
Tumor nodule number	
Solitary	85 (77.3%)
Multiple(≥2)	25 (22.7%)
Edmondson-Steiner grade	
I-II	46 (41.8%)
III-IV	64 (58.2%)
Microvascular invasion	
Absence	58 (52.7%)
Presence	52 (47.2%)
TNM stage	
Stage I	42 (38.2%)
Stage II	48 (43.6%)
Stage III	20 (18.2%)
BCLC stage	
BCLC A	88 (80%)
BCLC B	22 (20%)

Supplementary Table 2: The Cox regression analyses of overall survival (OS) and miR-425-5p expression level as well as clinicopathological parameters

Clinicopathologic Variables	n	Univariable analysis		Multivariable analysis	
		HR (95% CI)	*P*	HR (95% CI)	*P*
Gender					
Male	95	1		1	
Female	15	0.552 (0.220-1.382)	0.205	0.702 (0.238-2.072)	0.522
Age (years)					
≤ 60	85	1		1	
> 60	25	0.857 (0.444-1.655)	0.646	1.628 (0.754-3.517)	0.215
Liver cirrhosis					
Absence	89	1		1	
Presence	21	1.875 (0.887-3.964)	0.100	0.901 (0.391-2.077)	0.806
Liver function					
Child-Pugh B	101	1		1	
Child-Pugh A	9	0.143 (0.020-1.036)	0.054	0.170 (0.021-1.344)	0.093
Tumor size (cm)					
> 5	71	1		1	
≤5	39	0.836 (0.490-1.425)	0.510	0.719 (0.314-1.649)	0.436
Tumor nodule number					
Solitary	85	1		1	
Multiple(≥2)	25	2.269 (1.296-3.971)	**0.004**	0.426 (0.076-2.384)	0.331
Edmondson-Steiner grade					
I-II	46	1		1	
III-IV	64	1.153 (0.676-1.967)	0.601	0.960 (0.358-2.571)	0.935
Microvascular invasion					
Presence	58	1		1	
Absence	52	0.074 (0.036-0.153)	**<0.001**	0.030 (0.010-0.086)	**<0.001**
TNM stage					
Stage I	42	1		0.279 (0.122-0.639)	**0.002**
Stage II	48				
Stage III	20	2.356 (1.661-3.341)	**<0.001**	1	
BCLC stage					
BCLC A	88	1		1	
BCLC B	22	3.155 (1.800-5.531)	**<0.001**	11.711 (1.64-83.638)	**0.014**
miR-425-5p expression					
Low	55	1		1	
High	55	2.787 (1.611-4.822)	**<0.001**	2.857 (1.555-5.247)	**0.001**

Supplementary Table 3: The Cox regression analyses of disease-free survival (DFS) and miR-425-5p expression level as well as clinicopathological parameters

Clinicopathologic Variables	n	Univariable analysis		Multivariable analysis	
		HR (95% CI)	*P*	HR (95% CI)	*P*
Gender					
Male	95	1		1	
Female	15	0.601 (0.274-1.318)	0.204	0.819 (0.313-2.146)	0.685
Age (years)					
≤ 60	85	1		1	
> 60	25	0.855 (0.474-1.543)	0.603	1.013 (0.503-2.041)	0.970
Liver cirrhosis					
Absence	89	1		1	
Presence	21	1.922 (0.951-3.886)	0.069	0.885 (0.400-1.961)	0.764
Liver function					
Child-Pugh B	101	1		1	
Child-Pugh A	9	0.250 (0.061-1.022)	0.054	0.511 (0.113-2.302)	0.382
Tumor size (cm)					
> 5	71	1		1	
≤5	39	1.002 (0.607-1.654)	0.994	0.929 (0.441-1.956)	0.846
Tumor nodule number					
Solitary	85	1		1	
Multiple(≥2)	25	2.361 (1.394-4.000)	**0.001**	0.440 (0.072-2.674)	0.373
Edmondson-Steiner grade					
I-II	46	1		1	
III-IV	64	1.239 (0.756-2.030)	0.396	0.978 (0.404-2.365)	0.960
Microvascular invasion					
Presence	58	1		1	
Absence	52	0.021 (0.008-0.056)	**<0.001**	0.016 (0.005-0.051)	**<0.001**
TNM stage					
Stage I	42	1		1	
Stage II	48				
Stage III	20	2.737 (1.981-3.783)	**<0.001**	0.504 (0.218-1.163)	0.108
BCLC stage					
BCLC A	88	1		1	
BCLC B	22	3.576 (2.101-6.089)	**<0.001**	8.551 (1.077-67.87)	**0.042**
miR-425-5p expression					
Low	55	1		1	
High	55	2.703 (1.620-4.510)	**<0.001**	2.414 (1.394-4.180)	**0.002**
